# Effectiveness and safety of treating carotid atherosclerotic plaques with the method of nourishing qi, promoting blood circulation and expelling phlegm: A systematic review and meta-analysis

**DOI:** 10.3389/fphar.2022.1059737

**Published:** 2022-11-09

**Authors:** Jia Li, Yuying Du, Chao Cai, Fuming Liu

**Affiliations:** Affiliated Hospital of Nanjing University of Chinese Medicine, Jiangsu Province Hospital of Chinese Medicine, Nanjing, China

**Keywords:** carotid atherosclerosis, Chinese medicine, inflammation, meta-analysis, grade evaluation

## Abstract

**Objectives:** This meta-analysis aimed at evaluating the effectiveness and safety of Chinese medicine (TCM), which nourished qi, promoted blood circulation, and expelled phlegm (YQHXZT), in treating carotid atherosclerosis (CAS) from an immunological perspective.

**Background:** The incidence of CAS has been increasing and tends to be younger. Although western medicine is effective, there are some limitations. TCM has certain advantages over the multichannel and multitarget treatment strategies in slowing down the process of CAS. However, there is no comprehensive review in this field.

**Methods:** Nine databases were searched from January, 2012, to September, 2022. After applying the inclusion and exclusion criteria to the RCTs, research quality evaluation and data extraction were conducted, and a meta-analysis of the articles was performed. The GRADE was used to assess the quality of the evidence.

**Results:** Fourteen RCTs involving 1,191 patients were identified. The results indicated that the experimental group was more effective in improving carotid intima-media thickness (CIMT)[SMD = −0.97, 95%CI(−.30,−0.65), *p* < 0.00001], reducing carotid plaque area [SMD = −1.98, 95%CI(−3.06,−0.89), *p* = 0.0003], lowering hs-CRP [SMD = −1.33, 95%CI(−1.59,-1.06), *p* < 0.00001] and LDL-C levels [SMD = −0.60, 95%CI(−0.83,-0.38), *p* < 0.00001]. Moreover, the experimental group was superior to peak systolic blood flow velocity (PSV) [SMD = −0.37, 95%CI(−0.59,−0.16), *p* = 0.0007], clinical efficacy [RR = 1.64, 95% CI (1.39, 1.94), *p* < 0.00001] and plaque area efficacy [RR = 1.36, 95% CI (1.22, 1.52), *p* < 0.0001]. The adverse reactions were not statistically significant in the two groups [RD = -0.01, 95% CI (-0.04.0.01), *p* = 0.17]. The results of grade evaluation suggested that the outcome indicators LDL-C, hs-CRP, plaque area efficacy, PSV, and adverse events were moderate. CIMT, plaque reduction area, and TCM clinical efficacy were low-quality.

**Conclusion:** The combination of YQHXZT can alleviate the process of CAS by inhibiting the thickening of CIMT, reducing plaque area and lowering hs-CRP and LDL-C levels. The mechanism may possibly be related to reducing lipid deposition and inhibiting the inflammatory response. Besides, the combination did not increase the risk of adverse effects. However, more well-designed RCTs are needed in the future.

**Systematic review registration:** CRD42022360529, https://www.crd.york.ac.uk/prospero/

## 1 Introduction

Atherosclerosis-related diseases such as ischemic heart diseases and strokes have a high morbidity and mortality rate as well as a poor prognosis (Libby, 2021), putting a heavy physical and financial burden on patients (Humphries et al., 2018). Atherosclerosis (AS), as a high-risk factor and a major pathological basis for cardiovascular and cerebrovascular events (Prandoni et al., 2017), has become more prominent in modern decades as people’s living standards have enhanced, and the prevalence is expected to rise by 18% by 2030 (Valanti et al., 2021). AS is commonly classified as Carotid atherosclerosis sclerosis (CAS), Coronary atherosclerosis, lower limb atherosclerosis, and so on, depending on the location of the lesion. The carotid artery is one of the most commonly involved sites of systemic AS and is frequently used as a window into and assessment of the body’s large middle arteries, which can provide a general picture of systemic AS. According to studies, AS in the extracranial segment of the carotid artery is associated with the onset of cerebral infarction in 20%–25% of patients (Chen et al., 2019). Previous investigation (Karataş et al., 2016) has confirmed that the occurrence of AS is closely related to the inflammatory response mediated by inflammatory factors in the body, suggesting that the occurrence and progression of cerebral infarction may be related to the overexpression of inflammatory factors, dyslipidemia, and other factors. There is a critical need to address CAS prevention and treatment strategies.

In recent decades, both domestic and international researchers have identified AS a chronic inflammatory disease (Zhu et al., 2018). There is widespread agreement that inflammation plays a role in the development of AS (Geovanini and Libby, 2018). A growing number of clinical studies have attempted new anti-AS treatments based on anti-inflammation and immune regulation. Western medicine has had some success with lipid regulation, anti-inflammation, and anti-platelet aggregation. Nevertheless, there are some drawbacks to clinical application (Pang et al., 2017), such as muscle aches and pains, liver and kidney toxicity, increased transaminases (Backes et al., 2017), high drug production costs, the single target of the action, and so on (Li et al., 2020). As a result, research into CAS treatment is still a long way off. Traditional Chinese medicine (TCM) has been actively participating in the treatment of CAS in Asia and has demonstrated a definite curative effect with fewer side effects (Sun et al., 2021). TCM can affect multiple aspects of the occurrence and development of diseases from a holistic standpoint. TCM’s prevention and treatment of AS *via* immune inflammation-related pathways is a hot topic in current research. Extensive studies have discovered that qi deficiency and phlegm stasis are the most common types of evidence in CAS patients. The treatment of CAS primarily focuses on activating blood, resolving stasis, expelling phlegm, and benefiting qi (Wu et al., 2017), which shares similarities with western medicine in terms of lipid regulation and anti-inflammation. Randomized controlled trials (RCTs) on the clinical efficacy of AS have confirmed that Chinese medicines can reduce the size of CAS plaques and slow down the progression of CAS. However, few writers have drawn on any systematic study. The majority of RCTs were restricted to individual clinical observation and examination of a specific prescription. Most of the recommendations were self-recommended and based on empirical evidence, which was relatively weak. The safety and efficacy of these medications were not yet supported by scientific evidence due to the wide variety of Chinese medications, inconsistent dosage forms and doses, and some effective active ingredients of Chinese medications that had not undergone thorough pharmacological mechanisms of action studies. Given this, to aid the development of better clinical judgments, we thoroughly assessed the efficacy and safety of the YQHXZT treatment for CAS in this work.

## 2 Methods

### 2.1 Protocol and registration

The International Prospective Register of Systematic Reviews has received the protocol for this study (PROSPERO, CRD42022360529). The Preferred Reporting Items for Systematic Reviews and Meta-Analyses (PRISMA) (Moher et al., 2009) served as the foundation for this meta-analysis (Supplementary Table S1).

### 2.2 Search strategy

**FIGURE 1:**
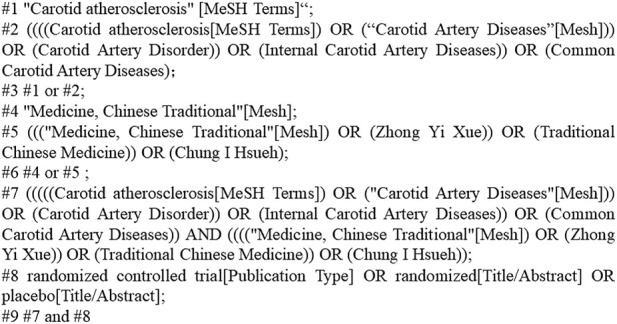
Search strategy.

We looked through PubMed, Embase, Cochrane Library, Web of Science, Medline, CNKI, VIP, Wan Fang, and CBM. The period of retrieval, which was only applicable to Chinese and English literature, ran from 1 January 2012, through 1 September 2022. The search was conducted using a combination of subject terms and free words. The Chinese search terms included “carotid artery atherosclerosis,” “qi deficiency and phlegm stasis,” “benefit qi and resolve phlegm,” “activate blood circulation and resolve blood stasis,” etc. English search terms included “carotid atherosclerosis,” “carotid artery disease,” “traditional Chinese medicine,” “randomized controlled trial,” “RCT,” etc., For unified management, the retrieved papers were loaded into Endnote X9. Figure 1 illustrated the precise search approach using PubMed as an example.

## 3 Eligibility criteria

### 3.1 Types of studies

A clinical randomized controlled trial (RCT), whether blinded or with allocation concealment.

### 3.2 Types of participants

Participants who met the Western diagnostic criteria for CAS as well as the Chinese medical evidence of Qi deficiency and phlegm stasis were tested. Gender, race, and disease duration were not limited, and the underlying disease was stable. The Diagnostic Criteria for Neurological Disorders served as the foundation for western medical diagnosis (Bai, 2009). The carotid ultrasound findings were consistent with the Vascular Ultrasound Guidelines’ relevant diagnostic criteria. Intima-media thickening was defined as IMT 1.0 mm; limited IMT 1.5 mm, projecting into the lumen, or limited IMT thickening above 50% of the peripheral IMT was defined as AS plaque formation. TCM diagnosis was based on “Guidelines for Clinical Research on New Chinese Medicines” and “National Standard of the People’s Republic of China-Chinese Medicine Clinical Diagnosis and Treatment Terminology, Evidence and Marks Part,” which were related to Qi deficiency and phlegm stasis. Headache, weakness, dizziness, chest tightness, shortness of breath, dullness, heaviness, and limb numbness are the most common symptoms. Palpitations, insomnia, pale mouth, fatigue, distention, poor appetite, localized tingling, pale or purplish face, pale or cyanotic lips, and cold limbs are secondary symptoms. Pale tongue with tooth marks or purple with petechiae, a thin, white, or slippery coating. The pulse can be slick or tight. If more than two of the primary symptoms listed above are present, or if one primary symptom coexists with two secondary symptoms, the diagnosis may be made by consulting the tongue and pulse signs.

### 3.3 Types of interventions

Both groups received standard basic therapies like hypotension and glucose reduction. Chinese medicine compound preparations (water decoction and granules only) were used in the test group, whereas lipid-regulating medications like atorvastatin and rosuvastatin were given to the control group.

### 3.4 Types of outcome measures

Carotid plaque morphological changes [carotid intima-media thickness (CIMT), plaque area], serum hypersensitive C-reactive protein (hs-CRP), and low-density lipoprotein (LDL-C) were the primary outcome indicators.

The effectiveness of TCM symptoms, the effectiveness of plaque area reduction, carotid hemodynamic parameters (peak systolic flow velocity, PSV), and the incidence of adverse events were all secondary outcome indicators.

### 3.5 Efficacy determination criteria

The book “Diagnostic and efficacy criteria for common diseases” should be read (Sun, 2010). Apparently effective: a 50% reduction in the size of the AS plaque. Effective: 20%–50% reduction in the size of the AS plaque. Ineffective: The area of the AS plaque was reduced by 20%.

According to the Guidelines for Clinical Research on New Chinese Medicines, clinical efficacy was classified into four levels of clinical control, apparently effective, effective, and ineffective, and symptoms were graded and scored as three for severe, two for moderate, one for mild, and 0 for asymptomatic. The total effective rate was determined by adding clinical control + apparently effective + effective. Clinical control: complete or partial disappearance of clinical symptoms and signs, as well as a 95% reduction in symptom score; apparently effective: significant improvement in clinical symptoms and signs, and a 70% reduction in symptom score; effective: improvement in clinical symptoms and signs, and a 30% reduction in symptom score; ineffective: no significant improvement in clinical symptoms and signs, or even aggravation, and a symptom score reduction of less than 30%. (pre-treatment points—post-treatment points)/pre-treatment points x 100% is the calculation formula.

### 3.6 Exclusion criteria

However, we excluded studies that had the following characteristics: non-clinical studies (animal cell experiments, systematic reviews, reviews, and empirical and medical cases); other therapies such as acupuncture and acupressure were also present in the intervention; outcome indicators did not include the outcome indicators observed in this study; inconsistent evidence patterns; inconsistent dosage forms; duplicate publications; lack of full text available; and tiny sample size.

### 3.7 Study selection

Two authors worked independently to screen the literature (Jia Li and Yuying Du). First, remove any duplicates. After scanning the study titles and abstracts to eliminate those that did not meet the criteria, a secondary screening was conducted using the inclusion and exclusion criteria after reading the full text of any studies whose status was unclear during the initial screening. In the event of a disagreement, a third assessor (Fuming Liu) joined the conversation and assisted with the decision.

### 3.8 Data extraction

The data extraction included broad details about the included studies, like the authors and the year of publication, as well as fundamental details of the studies, such as sample size and mean age; interventions and course; components of the risk of bias evaluation; and outcome indicators.

### 3.9 Quality assessment

Selection bias (random sequence generation and allocation concealment), implementation bias (blinding of the researchers and subjects), measurement bias (blinded evaluation of the study results), follow-up bias (completeness of outcome data), reporting bias (selective reporting of research results), and other biases. Each of the aforementioned entries can be categorized as either “low risk,” “unclear,” or “high risk,” with any discrepancies resolved by consulting with a third assessor.

### 3.10 Data synthesis and statistical analysis

A meta-analysis was performed using Review Manager 5.3 and Stata 12.1 software. Heterogeneity was analyzed using the χ2 test (test level *α* = 0.10), and if the heterogeneity between studies was more acceptable (*p* > 0.10 and I 2 ≤ 50%), a fixed effects model was selected for calculation; otherwise, a random-effects model was chosen. Standardized mean difference (SMD) for continuous variables and the risk ratio (RR) for dichotomous data with 95% confidence intervals (Cl) were used. The level of the meta-analysis was set at α = 0.05. When significant heterogeneity existed, subgroup analysis or sensitivity analysis was performed to find the source of heterogeneity, or only descriptive analysis was performed, depending on the data available. The publication bias was checked by funnel plots and Begg’s test if the number of trials was sufficient. When heterogeneity was detected, a sensitivity analysis was conducted to assess the stability of the results by excluding individual studies one by one. Subgroup analysis was performed to explore the sources of heterogeneity.

### Quality of evidence grade evaluation

The GRADE profiler 3.6 was applied to evaluate the outcome indicators, and the evidence levels were provided as high, moderate, low, and very low.

## 4 Results

### 4.1 Literature search

**FIGURE 2:**
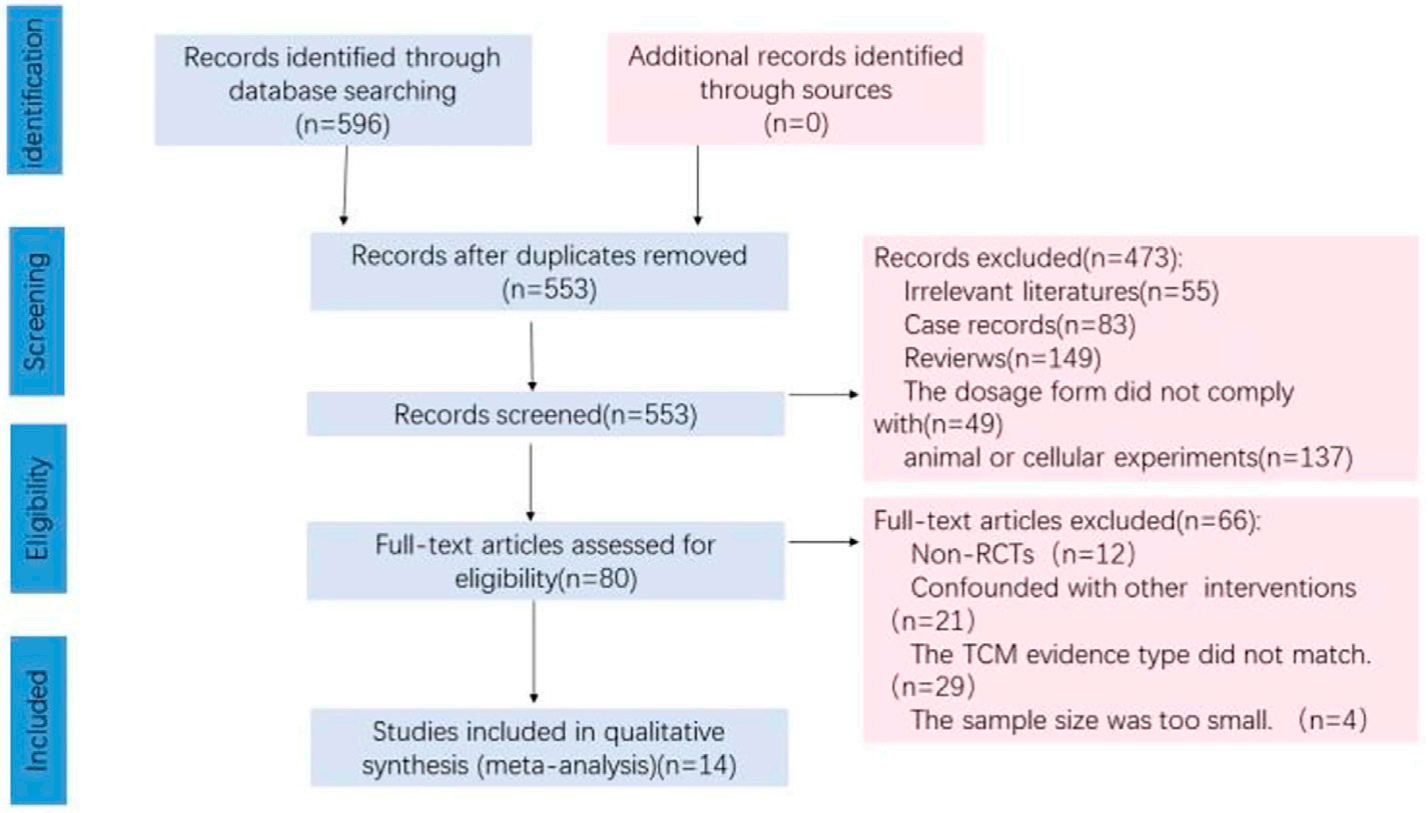
Flow diagram of the studies selection process.

A total of 596 studies were retrieved in the original screening. Forty-three duplicates were excluded, 473 of which were excluded after scanning the titles and abstracts. Moreover, sixty-six studies were further excluded after intensive full-text rescreening, and fourteen studies finally remained, all of which were in Chinese. The screening flow chart is shown in Figure 2.

### 4.2 Study characteristics and quality assessment

Fourteen studies were included in this study, involving 1,191 patients, 600 in the treatment group and 591 in the control group, with a sample size of 30–64 cases in the individual studies. The treatment group was the control group with the addition of the YQHXZT formula. The control group was Western medicine for lipid regulation. The duration of treatment lasted from 2 to 24 weeks. The basic characteristics of the 14 studies are detailed in Table 1.

**TABLE 1:** Basic characteristics of the included studies.

Study	Sample size (T/C)	Mean age (years, ‾x ± s)		Interventions		Treatment (weeks)	Ending indicators
		T	C	T	C		
Wu et al. (2018)	121 (62/59)	62.4 ± 3.7	61.9 ± 3.5	con + Yiqi huatan huoxue formula	Atorvastatin	24 weeks	①②③⑤⑧
Zhu (2018)	110 (55/55)	58.24 ± 2.96	59.52 ± 2.08	con + Yiqi huatan tongluo decoction	Rosuvastatin	24 weeks	②③⑤⑥⑧
Wang et al. (2019)	61 (31/30)	87.81 ± 4.34	89.10 ± 2.63	con + Yiqi huoxue huatan tongluo formula	Atorvastatin	12 weeks	④⑥⑧
Zhang and Guo (2015)	87 (44/43)	53.43 ± 8.17	52.45 ± 7.69	con + Yiqi huatan huoxue formula	Simvastatin	24 weeks	①②③⑥⑧
Cao and Wang. (2013)	120 (60/60)	57.5	55.6	con + Yiqi huatan huoxue formula	Rosuvastatin	24 weeks	②③⑤⑥⑧
Jiang (2019)	72 (36/36)	59.45 ± 9.879	57.97 ± 8.765	con + Xuemai shutong formula	Atorvastatin	12 weeks	②④⑥⑦⑧
Huang et al. (2012)	124 (64/60)	59.32 ± 7.77	57.27 ± 9.93	con + Liujun danshen granules	Atorvastatin	12 weeks	②⑥
Dong (2013)	60 (30/30)	83.93 ± 5.48	82.03 ± 6.74	con + Yiqi huatan huoxue formula	Simvastatin	24 weeks	②④⑥⑧
Zhang and Ye (2020)	60 (30/30)	49.74 ± 6.81	49.16 ± 6.37	con + Jiawei ditan formula	Atorvastatin	2 weeks	①②⑥⑦⑧
Hai (2017)	60 (30/30)	63.25 ± 9.15	62.15 ± 10.80	con + Jingling granules	Atorvastatin	8 weeks	②③④⑥⑧
Zhao et al. (2012)	76 (38/38)	66.4 ± 11.8	66.4 ± 11.8	con + Yiqi huoxue huatan tongluo decoction	Fluvastatin	16 weeks	②③④⑥⑦⑧
Zheng (2019)	80 (40/40)	74.96 ± 5.45	75.24 ± 5.63	con + Yiqi huoxue huatan tongluo formula	Simvastatin	12 weeks	②
Niu (2017)	80 (40/40)	52.49 ± 7.65	53.45 ± 8.15	con + Yiqi huatan huoxue formula	Simvastatin	24 weeks	①②③⑥
Chen (2020)	80 (40/40)	61.58 ± 8.86	59.68 ± 9.32	con + Buyang huanwu decoction	Atorvastatin	8 weeks	②④⑥⑦

Notes: T, intervention groups; C, control groups; con, control groups; ①PSV; ②CIMT; ③Carotid plaque area; ④Clinical efficacy of Chinese medicine; ⑤Plaque size efficacy; ⑥LDL-C; ⑦hs-CRP; ⑧Incidence of adverse reactions.

### 4.3 Literature bias and quality assessment

**FIGURE 3:**
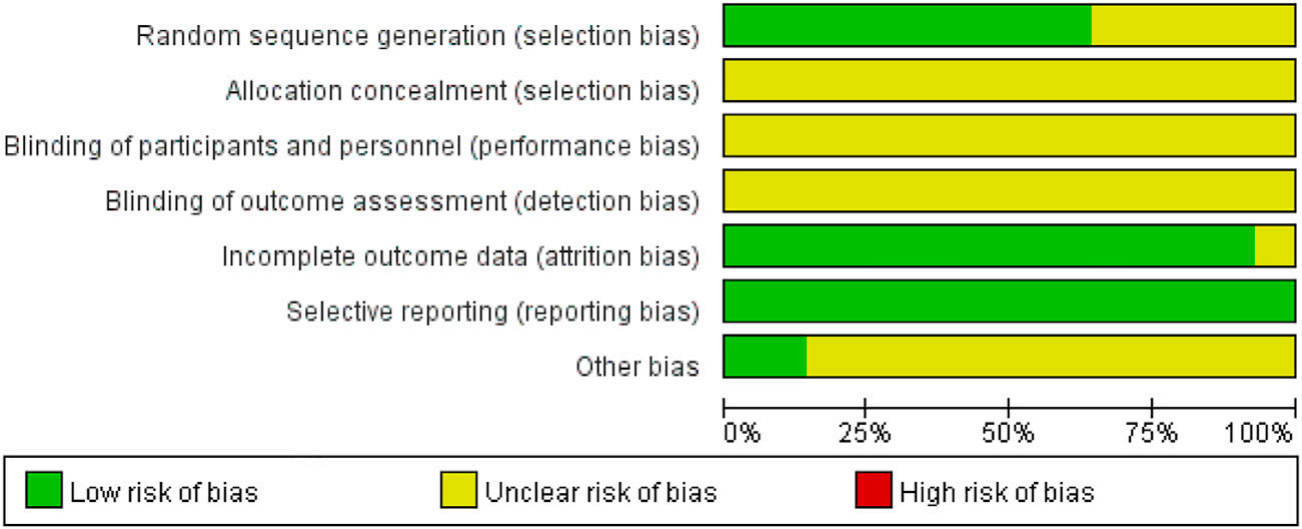
Risk-of-bias graph.

The quality of the included RCTs is shown in Figure 3. For the 14 included studies, all mentioned a randomized method of grouping, among which nine studies (Huang et al., 2012; Cao and Wang, 2013; Dong, 2013; Zhang and Guo, 2015; Zhu, 2018; Jiang, 2019; Wang et al., 2019; Chen, 2020; Zhang et al., 2020) described randomization methods in detail, such as the use of random number tables and the use of software randomized grouping, which was considered “low risk”; the rest of the studies only covered “randomization” without specifying the scheme. None of the studies mentioned assignment sequence hiding and blinding. There was no explicit description of blinding outcome raters. All studies included a balanced population at baseline with good data completeness. Pre-designed outcomes were reported in all studies, perceiving a low risk of selective reporting bias. Other biases were not found in all included studies. Two studies (Huang et al., 2012; Dong, 2013)were supported by government funding programs, had no conflicts of interest, and were otherwise assessed as “low risk” of bias; the source of the other bias for the remaining trials was unclear. The risk of bias for each study is shown in Figure 3.

## 5 Results of meta-analysis

### 5.1 Primary outcomes

#### 5.1.1 Morphological changes of carotid artery plaques

##### 5.1.1.1The thickness of the middle lining of the carotid artery (CIMT)

**FIGURE 4:**
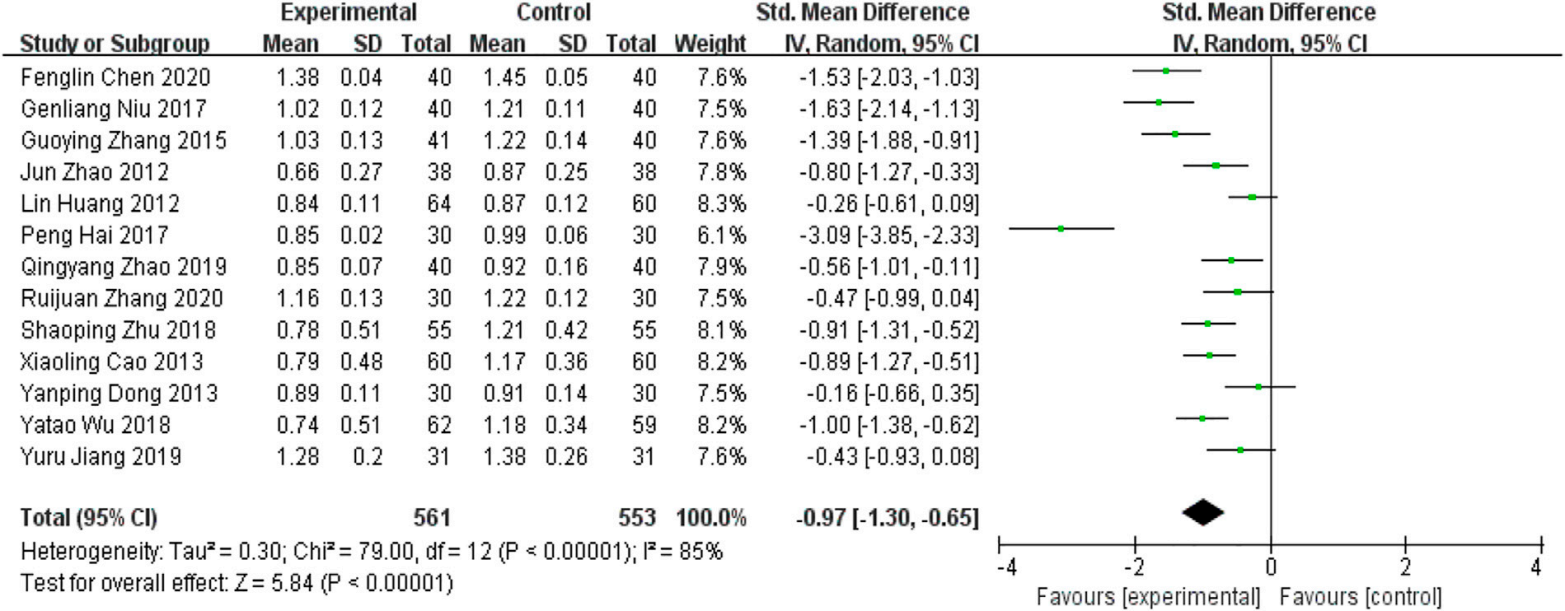
Forest plot for CIMT.

**FIGURE 5:**
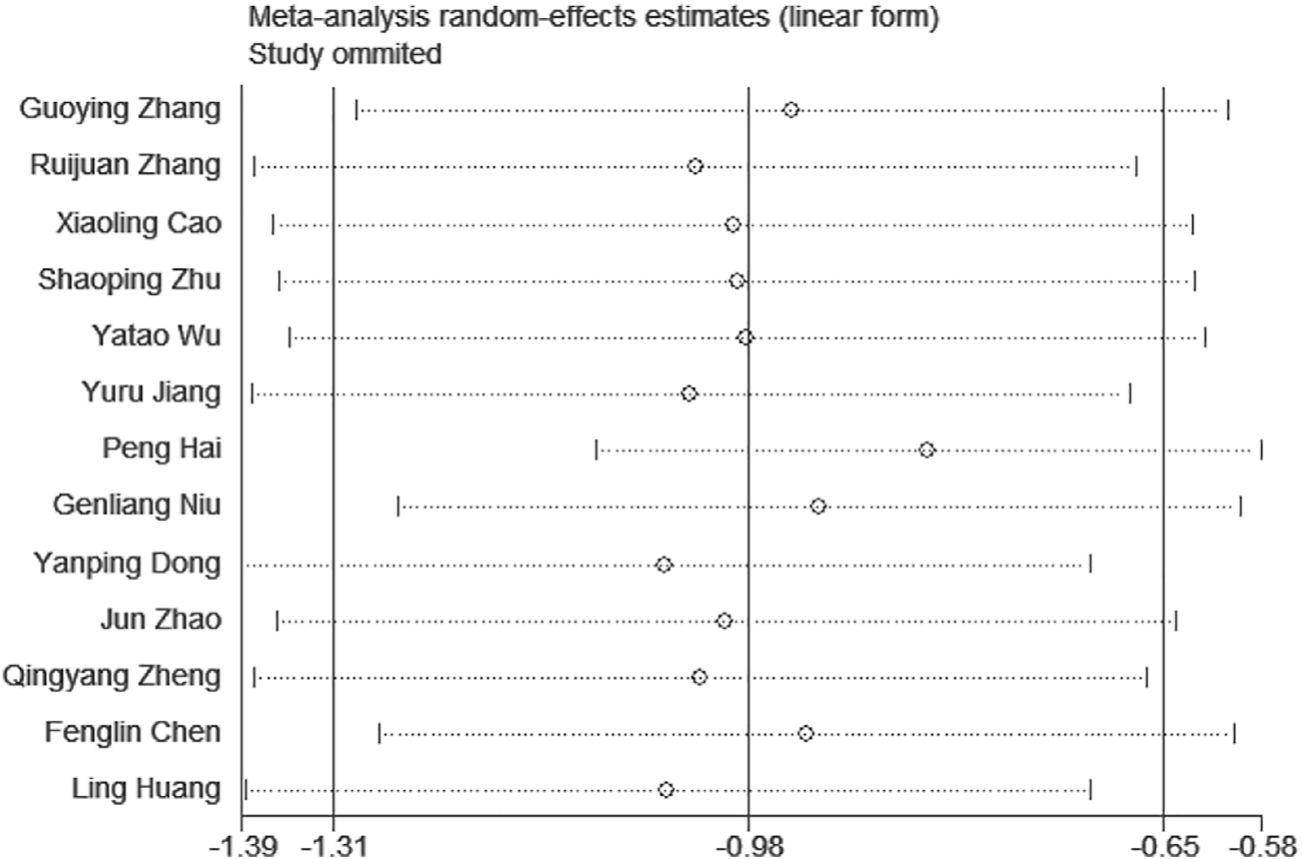
CIMT sensitivity analysis.

Pooled data from the thirteen studies (Huang et al., 2012; Zhao et al., 2012; Cao and Wang, 2013; Dong, 2013; Zhang and Guo, 2015; Hai, 2017; Niu, 2017; Wu et al., 2018; Zhu, 2018; Jiang, 2019; Zheng, 2019; Chen, 2020; Zhang et al., 2020) reporting the CIMT showed that YQHXZT formula clearly decreased the thickness of carotid medial intima as an adjuvant or monotherapy for CAS compared with the contrast group [SMD = -0.97, 95%CI (−1.30, −0.65), *p* < 0.00001; p for heterogeneity = 0.00001, *I* ^*2*^ = 85% > 50%; Figure 4]. The heterogeneity between studies was high, and the random effect model was selected for analysis. The Stata software was used for sensitivity analysis of the study, and no significant studies affecting the stability of the results were found (Figure 5).

The high heterogeneity was possibly related to the duration of treatment between studies. A meta-regression analysis of the duration of treatment was performed using Stata 12.1 software to find sources of heterogeneity. Three of the included studies had a treatment duration of <12 weeks and the remaining trials had a treatment duration of 12–24 weeks. The studies were divided into two groups according to treatment duration, and meta-regression was performed with the group (treatment duration) variable as a covariate, resulting in *p* = 0.101 > 0.05 and Adj R-squared = 17.43%, suggesting that the effect of treatment duration on heterogeneity was less likely and considered to be possibly related to the instrument used for measurement, the measurement researcher’s manipulation, measurement error, and other factors.

##### 5.1.1.2 Carotid plaque area

**FIGURE 6:**
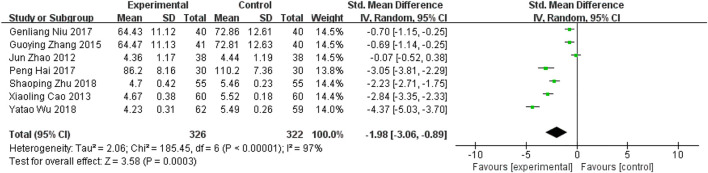
Forest plot for carotid plaque area.

Seven RCTs (Zhao et al., 2012; Cao and Wang, 2013; Zhang and Guo, 2015; Niu, 2017; Hai, 2017; Wu et al., 2018; Zhu, 2018) in the included studies reported carotid plaque area as an outcome indicator. Meta-analysis indicated that the addition of YQHXZT formula could significant further reduce the carotid plaque area [SMD = -1.98, 95%CI (−3.06, −0.89), *p* = 0.0003; p for heterogeneity *p* < 0.00001, *I* ^*2*^ = 97% > 50%; Figure 6]. Excluding each of the included studies one by one, no studies were found that significantly affected the stability of the results.

The reasons for the high heterogeneity were possibly related to factors such as duration of treatment and sample size across studies. A meta-regression analysis was performed using Stata 12.1 software to find sources of heterogeneity in terms of duration of treatment and sample size. A meta-regression was performed with the variables of group (duration of treatment (>12 weeks, <12 weeks) and sample size (>100 cases, <100 cases)) as covariates, and the results suggested that the factors of duration of treatment and sample size did not have a significant effect on heterogeneity (Table 2), and it was considered that they might be related to factors such as the instrument used to measure it, the operation of the researcher who measured it, and measurement error.

**TABLE 2:** Meta-regression analysis.

Factors	Regression coefficient	Coefficient standard error	t-value	*p*-value
Duration of treatment	−0.62	1.41	−0.44	0.68
Sample size	−2.04	0.94	−2.17	0.08

##### 5.1.1.3 Inflammatory factors— hs-CRP

**FIGURE 7:**
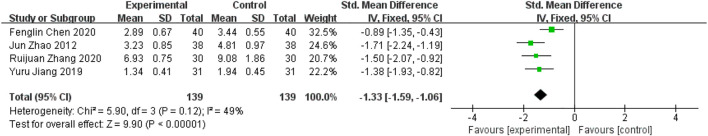
Forest plot for hs-CRP.

Four RCTs (Zhao et al., 2012; Jiang, 2019; Chen, 2020; Zhang et al., 2020) conducted hs-CRP as an inflammatory factor outcome. Meta-analysis revealed that the addition of YQHXZT formula was more likely to reduce hs-CRP levels [SMD = −1.33.95% CI (−1.59, −1.06), *p* < 0.00001, P for heterogeneity = 0.12, *I* ^*2*^ = 49% < 50%, Figure 7).

##### 5.1.1.4 Lipid levels— LDL-C

**FIGURE 8:**
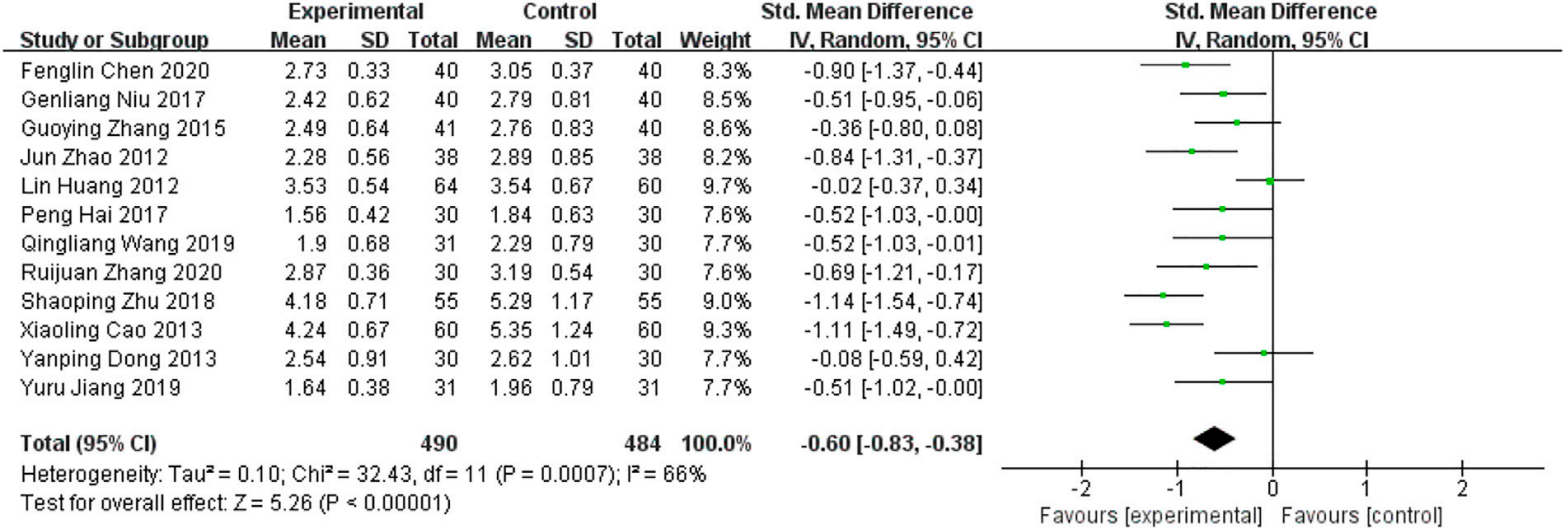
Forest plot for LDL-C.

**FIGURE 9:**
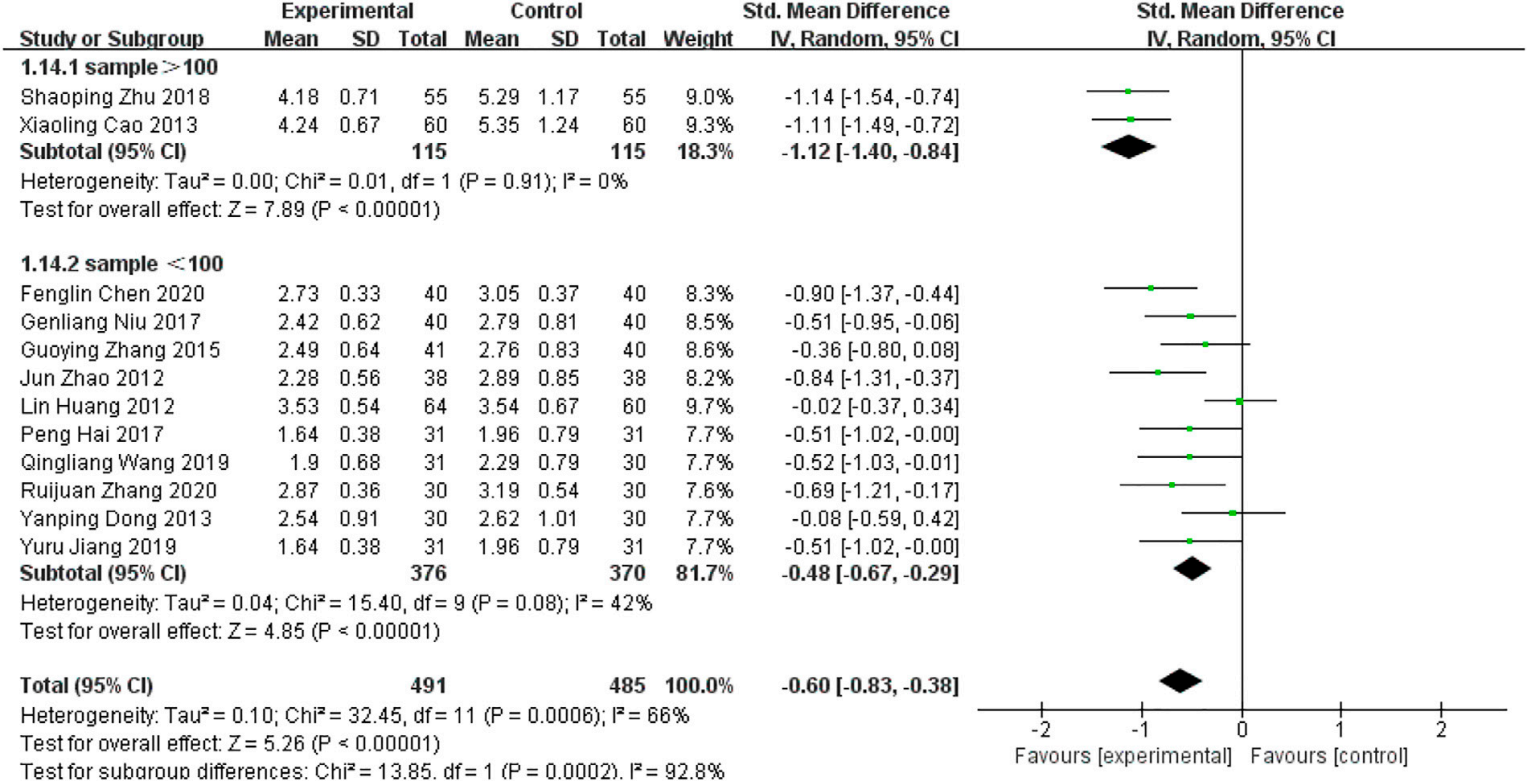
Forest plot for LDL-C subgroup analysis.

Twelve RCTs (Huang et al., 2012; Zhao et al., 2012; Cao and Wang, 2013; Dong, 2013; Zhang and Guo, 2015; Hai, 2017; Niu, 2017; Zhu, 2018; Jiang, 2019; Wang et al., 2019; Chen, 2020; Zhang et al., 2020) compared LDL-C levels before and after treatment in the two groups. From the data in Figure 8, it is apparent that the addition of the YQHXZT formula was clearly more effective in reducing LDL-C levels [SMD = -0.60.95%CI(−0.83,−0.38),*p* < 0.00001; P for heterogeneity = 0.0007, *I* ^*2*^ = 66% > 50%]. The results were analyzed by excluding each included study one by one, and no studies were found that significantly affected the stability of the results. The source of heterogeneity was possibly related to the sample size and a subgroup analysis was performed. Sample size >100, subgroup results showed that P for heterogeneity = 0.91, *I* ^*2*^ = 0% < 50%, *p* < 0.00001; sample size<100, subgroup results showed that P for heterogeneity = 0.08, *I* ^*2*^ = 42% < 50%, *p* < 0.00001, Figure 9. The heterogeneity of both subgroups was acceptable.

### 5.2 Secondary outcome indicators

#### 5.2.1 Carotid flow indicators——Peak systolic blood flow velocity

**FIGURE 10:**
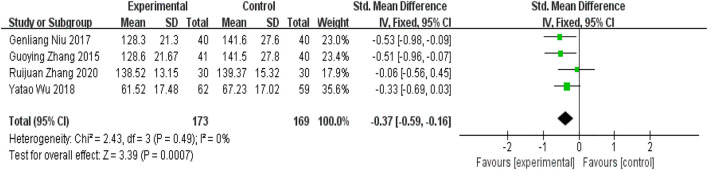
Forest plot for peak systolic blood flow velocity.

Four RCTs (Zhang and Guo, 2015; Niu, 2017; Wu et al., 2018; Zhang et al., 2020)used PSV as an outcome indicator. As Figure 10 showed, there was a significant difference between the two groups [SMD = −0.37, 95%CI(−0.59,−0.16),*p* = 0.0007; p for heterogeneity = 0.49 > 0.1; *I* ^*2*^ = 0% < 50%]. The experimental group was better at improving PSV.

#### 5.2.2 Efficacy indicators

##### 5.2.2.1 Clinical efficacy of Chinese medicine

**FIGURE 11:**
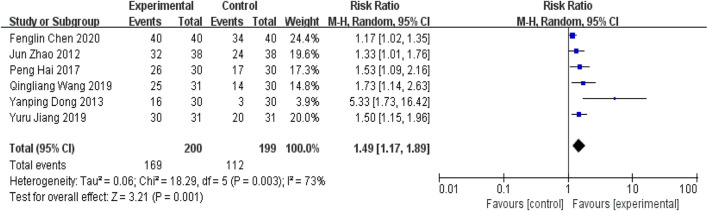
Forest plot for clinical efficacy of Chinese medicine.

**FIGURE 12:**
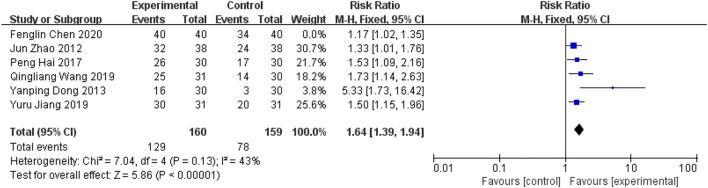
Forest plot of clinical efficacy for Chinese medicine excluding Chen.

Six RCTs (Zhao et al., 2012; Dong, 2013; Hai, 2017; Jiang, 2019; Ｗang et al., 2019; Chen, 2020) in the included studies used the clinical efficacy of TCM as an outcome indicator. Meta-analysis showed that the addition of YQHXZT formula was significantly more effective in improving the clinical outcome of TCM [RR = 1.49, 95%CI(1.17.1.89), *p* = 0.001,P for heterogeneity = 0.003, I^2^ = 73% > 50%, Figure 11]. Excluding each included study one by one, the heterogeneity was found to be reduced after removing the study by Fenglin Chen (Chen, 2020), *I* ^*2*^ = 43%, RR = 1.64, 95%CI (1.39, 1.94), *p* < 0.00001, the difference between the two groups was statistically significant (Figure 12).

##### 5.2.2.2 Plaque area efficacy

**FIGURE 13:**
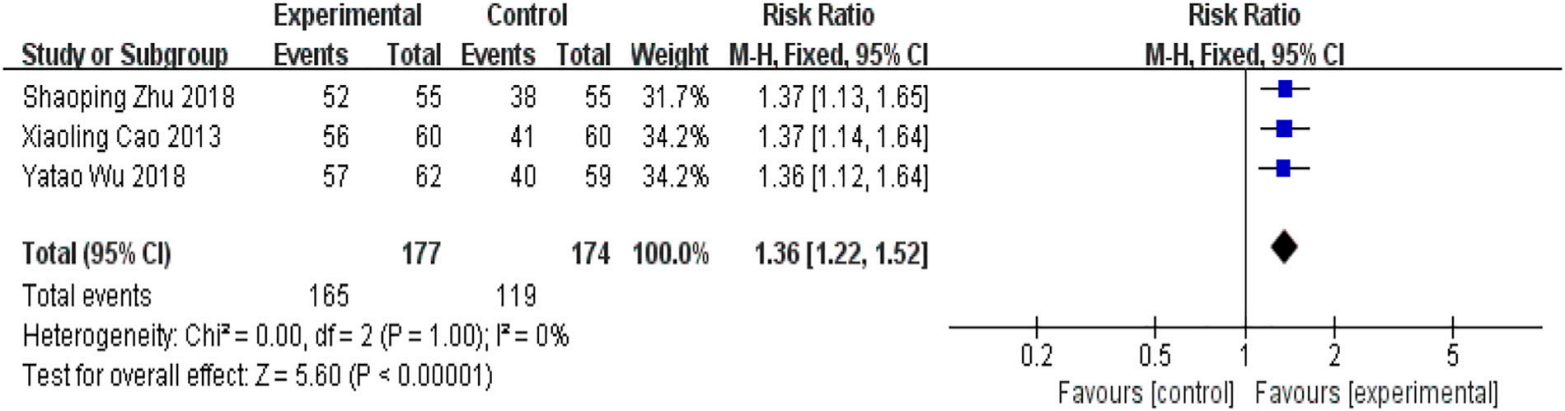
Forest plot for plaque area efficacy.

Three RCTs (Cao and Wang, 2013; Wu et al., 2018; Zhu, 2018) reported plaque area efficacy. The results of the meta-analysis were shown in Figure 13. The addition of the YQHXZT formula was superior to the control group in improving plaque size [RR = 1.36, 95%CI(1.22.1.52), *p* < 0.0001, P for heterogeneity = 1.00, *I* ^*2*^ = 0%].

##### 5.2.2.3 Safety indicators—Incidence of adverse reactions

**FIGURE 14:**
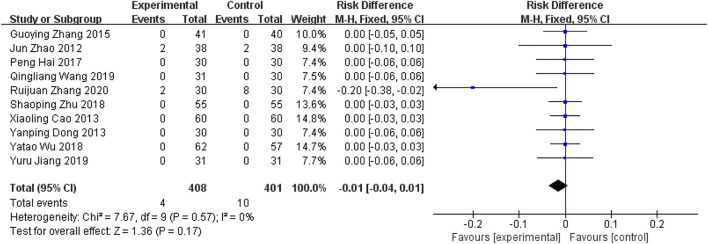
Forest plot of incidence for adverse reactions.

There were ten RCTs (Zhao et al., 2012; Cao and Wang, 2013; Dong, 2013; Zhang and Guo, 2015; Hai, 2017; Wu et al., 2018; Zhu, 2018; Jiang, 2019; Ｗang et al., 2019; Zhang et al., 2020) reporting incidence of adverse reactions as a safety indicator. Ruijuan Zhang (Zhang et al., 2020) reported adverse reactions dominated by nausea, rash, and liver function impairment in both groups, eight cases in the western medicine group and two cases in the trial group. Jun Zhao (Zhao et al., 2012) reported two cases of increased glutathione transaminase in each of the two groups, which returned to normal after discontinuation of the drug. There was no heterogeneity between the study results (*I* ^*2*^ = 0%) and a fixed effects model was used to calculate the combined statistic, which showed that the difference in the rate of adverse reactions between the experimental and control groups was not statistically significant [RD = −0.01, 95% CI (−0.04.0.01), *p* = 0.17, Figure 14].

##### 5.2.2.4 Publication bias

**FIGURE 15:**
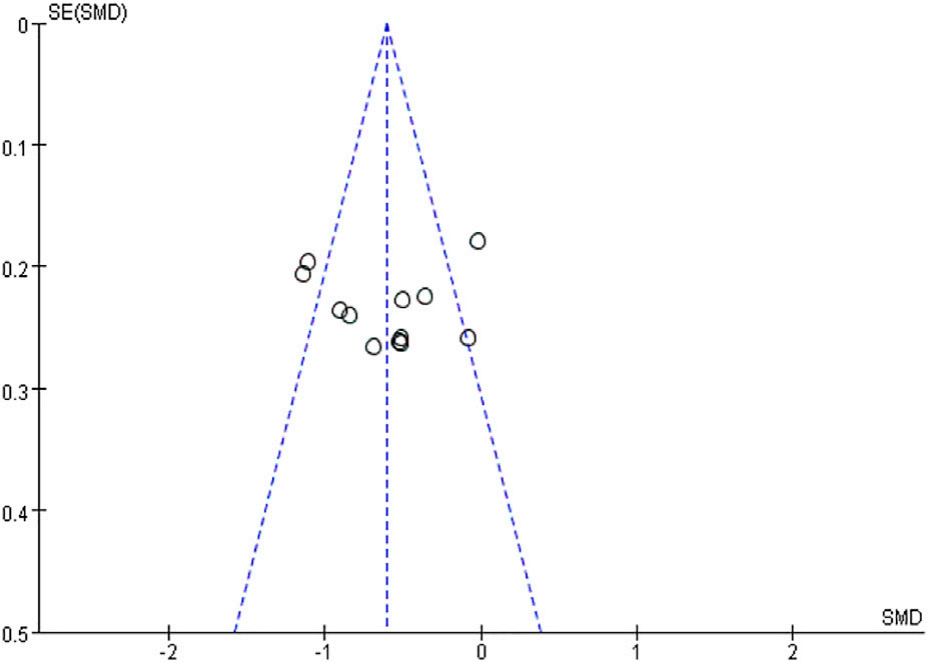
Publication risk of bias funnel plot.

**FIGURE 16:**
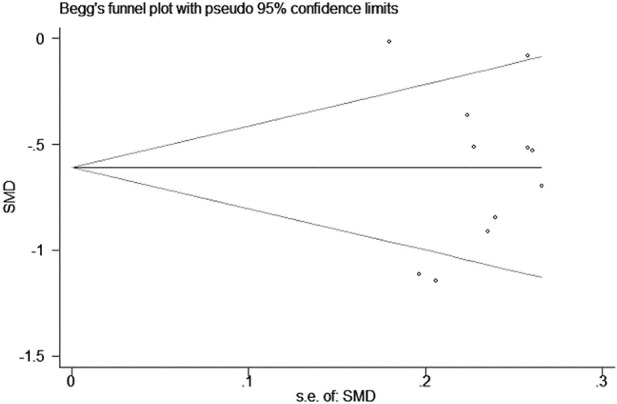
Graph of Begg’s test results.

The funnel plot showed a symmetric distribution of trials on either side of the funnel, and Begg’s test (*p* = 0.990 > 0.05) was consistent with the funnel plot, indicating that the results were reliable in this meta-analysis (Figures 15, 16).

### 5.3 Grade assessment

The evidence quality of outcomes was graded by using the GRADE system. The table below demonstrated that the outcome indicators PSV, LDL-C, plaque area efficacy, hs-CRP, and incidence of adverse events were moderate-quality evidence. The outcome indicators for CIMT, plaque area reduction, and clinical efficacy of TCM were of low-quality evidence. Relegation factors were mainly the absence of allocation protocol concealment and blinding and the high heterogeneity between studies (Table 3).

**TABLE 3:** Evaluation of GRADE evidence quality.

Outcome indicators	95%CI	Risk of bias	Inconsistency	Indirectness	Imprecision	Publication bias	Upgrade quality	Quality
CIMT	SMD −0.97 (−1.30, −0.65)	serious①	serious②	No	No	Undetected	None	Low
Carotid plaque area	SMD -1.98 (−3.06, −0.89)	serious①	serious②	No	No	Undetected	None	Low
hs-CRP	SMD −1.33 (−1.59, −1.06)	serious①	No	No	No	Undetected	None	Moderate
LDL-C	SMD −0.60 (−0.83, −0.38)	serious①	No	No	No	Undetected	None	Moderate
PSV	SMD −0.37 (−0.59, −0.16)	serious①	No	No	No	Undetected	None	Moderate
Clinical efficacy of Chinese medicine	RR 1.49 (1.17, 1.89)	serious①	serious②	No	No	Undetected	None	Low
Plaque area efficacy	RR 1.36 (1.22, 1.52)	serious①	No	No	No	Undetected	None	Moderate
Incidence of adverse reactions	RD −0.01 (−0.04.0.01)	serious①	No	No	No	Undetected	None	Moderate

SMD, is the standardized mean difference; RR, is the relative risk; ① absence of allocation concealment and blinding; ② the *p* value of the heterogeneity test was <0.1, and I^2^ >50%.

## 6 Discussion

The pathogenesis of AS is very complex. Inflammatory reactions not only run through all stages of the development of AS but also are closely related to the occurrence of various complications. The theory of immune inflammation has become a new breakthrough in the prevention and treatment of AS. The prevention and treatment of AS based on anti-inflammation and immune regulation have broad prospects and great potential. As one of the main blood supply vessels to the brain, the carotid artery plays an important role during AS. One study reported that the incidence of cerebral infarction in patients with greater than 70% carotid stenosis was approximately 13% (Zhang et al., 2020). There is no record of CAS in the Chinese medical literature. Based on its pathological nature, location, and clinical symptoms, it can be classified under the categories of “pulse paralysis”, “vertigo”, and “stroke.” Chinese medicine has recognized for thousands of years that the “veins” are the channels through which blood flows, and their physiological characteristics are similar to those of the “blood vessels” in Western medicine. In TCM, a qi deficiency is the root cause of the disease, and qi and blood are mutually beneficial. If qi is deficient, the blood is unable to move, and the blood is easily blocked and becomes stagnant. In addition, since the blood and fluid are of the same origin, when the arteries are blocked, the fluid stops, and the phlegm grows, the phlegm stagnates and adheres to the arteries, which turns into plaques over time and aggravates the progress of AS. In view of pathological factors such as phlegm, stasis, and turbidity, TCM clinically uses methods such as resolving phlegm, invigorating blood, and eliminating stasis to treat AS-related diseases (Jin et al., 2020). Statins are common clinical lipid-regulating drugs that can effectively improve lipid levels and plaque thickness and number in patients with CAS and reduce the incidence of adverse cardiovascular and cerebrovascular events (Liu et al., 2018). However, recently, investigators have examined the effects of western medicine alone are limited (Li, 2018), with shortcomings such as long duration, easy drug resistance, and side effects. In recent decades, TCM has received increasing attention due to its therapeutic effect and prospective future (Zhang et al., 2022). Previous research has established that the treatment of CAS using the YQHXZT method can effectively reduce intima-media thickness and plaque area, improve arterial stenosis, and enhance blood flow velocity (Chen and Zhang, 2017).

This study was based on Cochrane systematic evaluation principles to systematically evaluate the efficacy and safety of the combination of the YQHXZT method with western medicine in the treatment of CAS, and to evaluate the quality of evidence for the outcome indicators of the included studies by Grade profiler 3.6. As can be seen from above, the experimental group was more effective in improving CIMT [SMD = −0.97, 95%CI (−1.30, −0.65), *p* < 0.00001], reducing carotid plaque area [SMD = −1.98, 95%CI(−3.06,−0.89),*p* = 0.0003], lowering hs-CRP levels [SMD = −1.33, 95%CI(−1.59,−1.06),*p* < 0.00001] and LDL-C levels [SMD = −0.60, 95%CI(−0.83,−0.38), *p* < 0.00001]than the control group. Moreover, the experimental group was superior to PSV [SMD = -0.37, 95%CI(-0.59,-0.16), *p* = 0.0007]. In terms of clinical efficacy, the Chinese medicine clinical efficacy [RR = 1.64, 95%CI(1.39.1.94), *p* < 0.00001] and improvement of plaque area efficacy [RR = 1.36, 95% CI (1.22, 1.52), *p* < 0.0001] were both better in the experimental group. The incidence of adverse reactions was not statistically significant in the two groups [RD = −0.01, 95% CI (−0.04.0.01), *p* = 0.17]. The results of this review, combined with the grade evaluation, suggested that the outcome indicators LDL-C, hs-CRP, plaque area efficacy, PSV, and incidence of adverse events were moderate evidence, and the outcome indicators CIMT, plaque reduction area, and TCM clinical efficacy were low-quality evidence.

**FIGURE 17:**
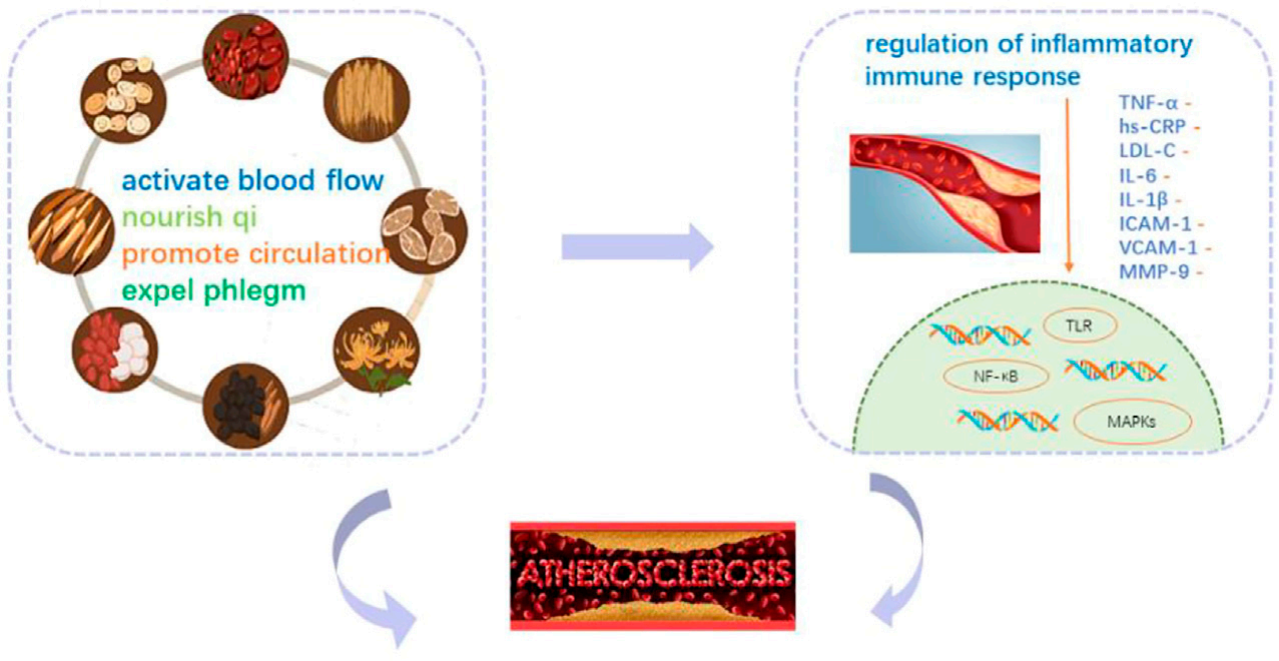
Mechanism of YQHXZY in improving CAS.

AS is a chronic vascular inflammatory response caused by the accumulation of LDL-C and is higher in patients with CAS. Within a certain range, the risk of AS increases logarithmically with higher LDL-C (Chen, 2020). The protein components of LDL particles in the body stimulate T lymphocytes to produce pro-inflammatory cytokines, which aggregate inflammatory factors such as CRP, IL-6, and monocyte chemoattractant protein-1, causing macrophages to phagocytose oxidized LDL and convert it into foam cells, which accumulate and eventually lead to the formation of AS (Beverly and Budoff, 2020). The vascular inflammatory response plays a key role in the pathogenesis of AS (Nie et al., 2020), influencing plaque formation, progression, and stability. Hs-CRP is a sensitive inflammatory factor reflecting vascular inflammatory mechanisms, which can be involved in related gene regulation and is closely related to the formation and breakdown of AS (Chen et al., 2021a). Hs-CRP can induce inflammatory and adhesion factors and make them accumulate under the vascular endo cortex, promote the formation of foam cells, and promote interstitial degradation and vascular smooth muscle cell apoptosis by activating the inflammatory pathway. This increases the risk of AS and plaque rupture. Tigkiropoulos et al. (Tigkiropoulos et al., 2019)demonstrated that PSV could be used to assess the degree of arterial stenosis. Studies suggested that every 0.1 mm thickening of the IMT increased the risk of infarction by 10%–15% and stroke by 13%–18%, especially in unstable plaques, which were more likely to dislodge and lead to stroke (Liu et al., 2020). Research has confirmed Chinese medicine’s effectiveness as an effective means of treating cardiovascular diseases (Hao et al., 2017). For example, Huangjing can play a role by regulating lipid metabolism, reducing inflammation, and improving oxidative stress (Chen et al., 2021b). Data from several studies suggest that increasingly Chinese medicine prescriptions, including Chinese patent medicine, classical prescription decoction, self-made prescription, etc., have been confirmed to regulate immune inflammation and antioxidant in a variety of ways (Tang et al., 2022). According to the composition and compatibility effect of the prescription, the most common is to benefit qi, promote blood circulation, and eliminate phlegm. The main pathways involved include Nf-κB pathway, the MAPKs pathway, the Toll-like receptor pathway, the inflammatory body pathway, etc (Figure 17). More attention has been paid to the first two pathways, whose mechanisms may be mainly by inhibiting NF-κB protein phosphorylation or NF-κB p65 gene expression and can also be combined with the MAPKs pathway to regulate the expression of related inflammatory cytokines to play an anti-inflammatory role. For example, berberine, the main compound of Huanglian, can lower blood lipid, antioxidation, anti-inflammation, and protect vascular endothelium (Xu et al., 2019) by downregulating the serum inflammatory factor levels, inhibiting MAPK phosphorylation, inhibiting NF-κBp65 mRNA expression (Feng et al., 2017).

In terms of publication bias, the funnel plot and Begg’s test were used to prove that there was no obvious publication bias in this study, and that the study results were reliable. In clinical work, it was found that AS was often combined with other diseases at the same time. According to the existing literature reports, TCM could also improve some symptoms and indicators of concomitant diseases, but its efficacy still needs further standardized study. This study, combined with grade evaluation, provides an evidence-based basis for clinical treatment direction. Compared with the control group, the YQHXZT formula was more comprehensive and safer, which was worthy of further clinical promotion.

## 7 Conclusion

This study has shown that the combination of the YQHXZT method could inhibit carotid intimal thickening, reduce plaque area, lower LDL-C and hs-CRP levels, and improve clinical efficacy. At the same time, the combination of YQHXZT methods has not found a significant increase in the risk of adverse reactions. Overall, this study probably strengthens the idea that the combination of Chinese medicine and western medicine can slow down the CAS process and improve the prognosis of the disease by inhibiting the inflammatory response and regulating lipid metabolism. That provides new attempts for the treatment of CAS in the future.

## 8 Limitation

However, this study also had some limitations: ① Some of the included studies did not describe the stochastic method in detail. No trial mentioned allocation concealment and blinding, and selection bias may have occurred. The included studies were Chinese studies with insufficient research dimensions. ② Mostly limited to the observation and analysis of a prescription, should focus on some good curative effects of YQHXZT by TCM fixed prescription or Chinese patent medicine. Therefore, in the future, we should pay attention to the improvement of methodology and carry out multicenter, large sample, high-quality, long-course double-blind RCT with Chinese medicine characteristics for verification. At the same time, the basic research on the treatment of CAS with TCM was relatively weak, and the research on the mechanism of action of drugs needed to be more in-depth and clearer (Yang et al., 2020).

## Data Availability

The raw data supporting the conclusion of this article will be made available by the authors, without undue reservation.

## Abbreviations

YQHXZT, nourishing qi, promoting blood circulation, and expelling phlegm; CAS, carotid arteriosclerosis; AS, arteriosclerosis; RCTs, Randomized controlled trials; TCM, Traditional Chinese medicine.
